# Selective Breeding for a Behavioral Trait Changes Digit Ratio

**DOI:** 10.1371/journal.pone.0003216

**Published:** 2008-09-17

**Authors:** Reginia H. Y. Yan, Jessica L. Malisch, Robert M. Hannon, Peter L. Hurd, Theodore Garland

**Affiliations:** 1 Department of Psychology, University of Alberta, Edmonton, Alberta, Canada; 2 Department of Biology, University of California Riverside, Riverside, California, United States of America; Lund University, Sweden

## Abstract

The ratio of the length of the second digit (index finger) divided by the fourth digit (ring finger) tends to be lower in men than in women. This 2D∶4D digit ratio is often used as a proxy for prenatal androgen exposure in studies of human health and behavior. For example, 2D∶4D ratio is lower (i.e. more “masculinized”) in both men and women of greater physical fitness and/or sporting ability. Lab mice have also shown variation in 2D∶4D as a function of uterine environment, and mouse digit ratios seem also to correlate with behavioral traits, including daily activity levels. Selective breeding for increased rates of voluntary exercise (wheel running) in four lines of mice has caused correlated increases in aerobic exercise capacity, circulating corticosterone level, and predatory aggression. Here, we show that this selection regime has also increased 2D∶4D. This apparent “feminization” in mice is opposite to the relationship seen between 2D∶4D and physical fitness in human beings. The present results are difficult to reconcile with the notion that 2D∶4D is an effective proxy for prenatal androgen exposure; instead, it may more accurately reflect effects of glucocorticoids, or other factors that regulate any of many genes.

## Introduction

Researchers have long noted that men tend to have lower index∶ring finger ratios than women [1], [2], [3]. Anecdotal reports of subtle differences in such measures of hand shape between mathematicians and engineers, as compared with artists and litterateurs, for example, date back at least a century [4]. This 2D∶4D ratio is lower, more masculine-typical, in both men and women of greater physical fitness, or who participate at more elite levels of athletic competition [5]–[10]. 2D∶4D has been proposed to correlate with health and personality traits because of the common organizational influence of prenatal testosterone across these traits [11]–[14]. Although studies linking prenatal environment to variation in digit ratio do imply a role for fetal testosterone, a direct cause-and-effect relation is far from proven. For example, girls with congenital adrenal hyperplasia have lower, more male-like digit ratios [15], [16]. Congenital adrenal hyperplasia involves disruption of the glucocorticoid synthesis pathway in the adrenal glands and leads to an absence of circulating glucocorticoids, triggering an over-production of androstenedione, which in turn results in abnormally high adrenal androgen production. The ratio of testosterone to estradiol in amniocentesis samples collected in the second trimester correlated with the child's 2D∶4D ratio at two years of age in a sample of 18 boys and 15 girls [17]. Female dizygotic twins having male co-twins have more masculine left hand 2D∶4D than those with female co-twins [18]. Similarly, mice that gestate next to males have larger ratios than those gestating next to females [19], suggesting an effect of fetal testosterone in the opposite direction. Behavioral studies correlating 2D∶4D in humans and mice also suggest similar, but opposite effects: humans with lower 2D∶4D show greater propensity for aggression [20]–[22], whereas inbred mouse strains with larger 2D∶4D were more likely to bite while being handled [23]. Lower digit ratios are associated with greater physical fitness or athletic competitiveness in men and women [5]–[10], but mouse strains with higher digit ratios tend (p = 0.058) to show higher daily activity levels (measured by the total number of beam breaks per day in an individual live-in cage) [23].

The overwhelming trend in human digit ratio studies shows more masculinized 2D∶4D to be associated with more masculinized behavior within each sex, so the studies linking sporting achievement and digit ratio make intuitive sense. However, the idea that 2D∶4D simply reflects the organizational effects of testosterone cannot be the whole story. The emphasis on prenatal testosterone as the major determinant of digit ratio runs counter to the fact that variation in digit ratio among human ethnic groups is far larger than differences between the sexes [24]–[26]. Similarly, lab mice show large inter-strain differences in 2D∶4D [23], but evidence for a sex difference in lab mice is mixed: two small studies show a higher 2D∶4D in females [27], 28, while a larger study finds no difference [23]. Whether digit ratio in mice is sexually dimorphic, and whether variation in rodent digit ratio can serve as a reliable proxy for prenatal environment in behavioral studies, is a matter of debate [29], [30]. Some [e.g. 11] have argued that the digit ratio effect is driven by regulation of HOX genes by circulating androgens. Evidence of digit ratio effects from other taxa have been ambiguous. In lizards, sex differences have been reported in opposing directions for different species within the same study [31]. Studies of *Anolis carolinensis* have reported significant effects of sex as well as significant differences between laboratories/populations [32], but another smaller study reports no significant difference [33]. Similar patterns of results have been reported in birds, where sex differences found in zebra finches [34] were not replicated by a larger study [35], and in pheasants where sex differences in 2D∶4D were found in one population [36], but not another [37]. Given the highly conserved nature of HOX genes, and their pleiotropic effect on morphological development of both gonads and fingers, one ought to expect a highly robust sex effect on digit ratio across taxa [28].

We used an experimental evolution approach [38] to investigate the link between behavior and digit ratios in laboratory house mice. We compared mice from lines that have been selectively bred for high rates of voluntary exercise with their unselected control lines. Mice from the selected (High Runner, HR) lines exhibit many differences relative to control lines, including higher voluntary wheel-running speeds, home-cage activity, maximal aerobic capacity, and predatory aggression, but no difference in maternal or intermale aggression [39]–[42]. Importantly, the HR lines also show 2-fold elevated baseline circulating corticosterone, but no apparent difference in circulating testosterone [40], [42], [43]. We hypothesized that, if digit ratio reflects the organizational effects of testosterone, then we would find a consistent sex difference in the mice. The digit-ratio-reflects-testosterone hypothesis predicts no difference between HR and control lines, given the absence of an effect of the selective-breeding regime on testosterone levels. We found a sex difference, higher 2D∶4D in females than males, statistically significant in the selected lines only, and an effect of selective breeding, higher 2D∶4D ratios in HR lines, in both sexes. The latter effect seems to contradict human studies, where masculinized digit ratios are associated with greater physical fitness or sporting achievement.

## Results

**Figure 1:**
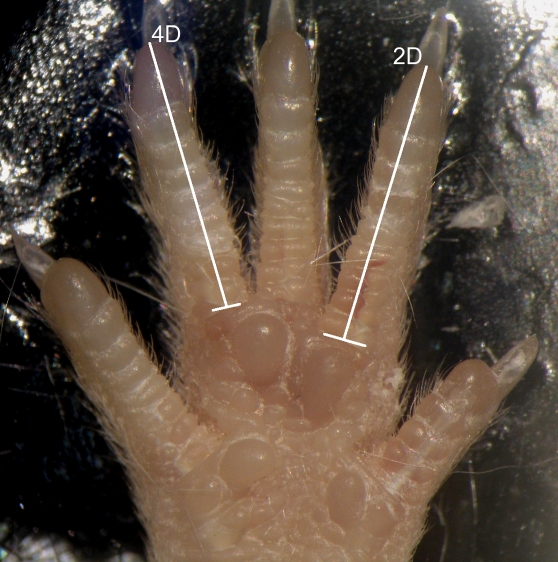
Measurement of digit lengths. Right rear palm of male mouse from a line bred for high running, illustrating digit lengths, measured from the mid-point of the basal crease to digit tip. The 2D∶4D ratio was 573.6∶585.0 pixels, yielding a 2D∶4D of 0.9805.

We compared hind limb 2D∶4D, measured from the mid-point of the basal crease to digit tip (Fig. 1), of the four HR lines (male∶female N = 66∶79, 74∶89, 69∶62, 77∶69) with those of the four Control (C) lines (N = 48∶61, 49∶54, 58∶58, 53∶47).

**Figure 2:**
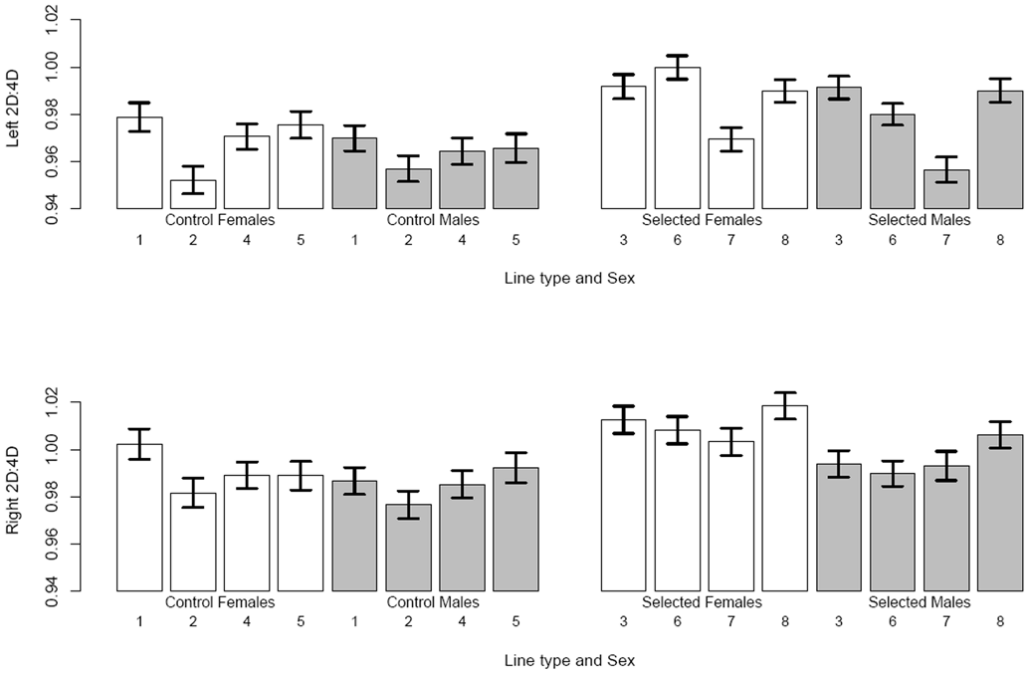
Digit ratios by sex and selection line. 2D∶4D finger length ratios by sex and selection regime for the eight lines of mice. Control are lines 1, 2, 4 and 5 (lab designations), High Runner selected lines are 3, 6, 7 and 8), open bars indicate females, and shaded bars males. Values are least square means and associated standard errors from models (separately for Control and Selected lines) including sex, line, sex-by-line interaction, and generation as fixed effects (SAS Procedure Mixed: family nested within line and the sex-by-family(line) interaction were random effects). See text for P values and for results of analyses comparing the four Control with the four High Runner lines.

Overall, 2D∶4D was higher in females than males, and in HR lines than Control lines (Fig. 2). On the right limb, differences were significant for sex (F_(1,6)_ = 12.88, p = 0.0115) and linetype (F_(1,6)_ = 11.76, p = 0.0140), without significant interaction between these main effects (F_(1,6)_ = 2.99, p = 0.1345) (for the random effects, χ^2^ = 5.10, d.f. = 3, P = 0.0779). Results were similar on the left limb, but did not reach statistical significance (sex F_(1,6)_ = 4.88, p = 0.0691; linetype F_(1,6)_ = 4.71, p = 0.0730; interaction F_(1,6)_ = 0.19, p = 0.6803) (for the random effects, χ^2^ = 37.11, d.f. = 3, P<0.0001). The finding of significant effects only on the right side fits the general pattern of previous research on human beings [11], [12].

Although the interaction between sex and linetype was not statistically significant, Figure 2 suggests that the significant sex effect on the right paw is driven by the HR lines. Indeed, separate analyses of the Control and HR lines showed no sex difference for either limb in Control lines (right F_(1,91)_ = 1.63, p = 0.2047; left F_(1,91)_ = 1.76, p = 0.1879), whereas HR lines showed a sex difference in 2D∶4D on both rear limbs (right F_(1,81)_ = 14.56, p = 0.0003; left F_(1,81)_ = 5.74, p = 0.0189).

## Discussion

Selective breeding for the behavioral trait of high voluntary exercise (wheel running) raised the 2D∶4D digit ratio in these mice, suggesting that the selection regime “feminized” them, as females in this study had larger 2D∶4D ratios than males. The direction of the sex effect, larger in females than in males, agrees with those studies that have found sex differences in mice [27], [28]. Similarly, the effect of selective breeding parallels the trend among inbred strains where higher total daily activity correlates positively with digit ratio [23]. Therefore, the present results seem to contradict the human studies, where masculinized digit ratios are associated with greater physical fitness or sporting achievement.

Several possible explanations exist for this discrepancy, the first being that the HR lines are indeed the more feminized, but that high voluntary wheel running in mice is not a homologous trait to the traits assayed in the human studies. Female mice run more than males [42]. In human studies, the physical fitness measures used can be broadly classified as either level of competition in hierarchically organized sports [5]–[7] or objective performance scores on athletic tasks [8]–[10]. In a study of an otherwise unselected sample of 77 women and 102 men (mostly university students) [8], the number of hours of exercise a week was measured, as a potentially confounding variable, and correlated significantly, and positively, with digit ratio (females, left hand: r = 0.27, P = 0.017; males, right hand: r = 0.38, P = 0.0001; note that these sex-by-hand combinations were the only ones correlating with physical fitness in the original study). This suggests that voluntary exercise in humans, unlike physical fitness, is associated with less masculinized 2D∶4D. Here, we found no such relationship in mice. In any case, the literature linking masculinized 2D∶4D with greater physical fitness in not unequivocal; for example, lower 2D∶4D is associated with earlier age of first myocardial infarction in men [28].

A second possible explanation is that higher exercise rates are in fact more “masculinized,” but that lower digit ratios do not indicate greater masculinization in these mice. The present data do not provide unequivocal support for larger 2D∶4D in females. The unselected control lines, like those in [23], do not show statistically significant sexual dimorphism. Other evidence, such as intrauterine position [19], suggests an effect in the opposite direction, higher in more masculinized individuals. On balance, the lack of any sex effect in that direction suggests strongly that if digit ratio indicates masculinization, then the high-running lines are the more feminized, and the possibility that lower digit ratios are more masculinized is a poor candidate explanation for our results.

A third possible explanation for our results is that propensity for voluntary physical exercise and 2D∶4D are not linked to masculinization or, if they are, not through prenatal androgen exposure. Although 2D∶4D was higher in high-running lines of mice, they do not have higher testosterone, at least as adults [40]. However, the sex and selection regime effects on 2D∶4D match those for adult baseline circulating corticosterone: females have higher corticosterone levels than males, and HR lines have higher levels than Controls [42], [43]. Similarly, females have higher 2D∶4D than males, and HR have higher 2D∶4D than Controls. Although increased circulating glucocorticoids are generally thought to depress testosterone, some evidence suggests that they are positively correlated in the human fetus [44]. We are unaware of any published work directly examining the effects of glucocorticoids on digit ratio. However, experimental manipulations of hormones other than testosterone have been demonstrated to influence digit ratio. Male, but not female, ring necked-pheasants show decreased right limb 2D∶4D in response to increased egg estradiol [36], whereas they show an increase in left limb 2D∶3D–not 2D∶4D–in females, but not males, in response to increased egg testosterone [37]. Given the many factors that have the ability to affect digit ratio, it is clearly more complicated than a simple testosterone-driven manliness metric. The significant additive genetic effects on human digit ratios imply a possible common effect of genes on both 2D∶4D and behavioral traits, aside from any common hormonal influence [44], [45]. Our results cast some doubt on 2D∶4D serving as an indicator of prenatal testosterone exposure, per se. Nonetheless, our results support the idea that relative digit length reflects some endocrinology-related aspect of development. Further investigation of the interacting effects of genetic variation, perinatal hormone levels, and such environmental influences as prenatal maternal stress and perinatal maternal care will be required before the full story is understood.

## Materials and Methods

The selective breeding methodology is described in [39]. To achieve a large sample size (total N = 1,013), mice were sampled from generations 45–48. Digits were measured by RHY (blind to generation, sex, line, and linetype), following the same procedures as in our previous work [13], [14]. Briefly, hind paws preserved in 4% paraformaldehyde were straightened and fixed palm-up to an adhesive backing, then photographed via stereoscope. Digit length was measured from the mid-point of the basal crease to digit tip (Fig. 1). Each paw was photographed and measured twice while the paw was left in the same position. The mean value of the two measures (which were highly correlated, Pearson's r = 0.9 for both left and right paws), was used for analysis. To calculate the reliability of the 2D∶4D measures, we re-imaged a random sample of 50 mice several months after the original images were taken, then calculated the intraclass correlation coefficient following [47]. Left paws: ICC = 0.66 (95%CI: 0.472<ICC<0.792), F(49,50) = 4.89, P≪0.001), Right paws: ICC = 0.544 (95%CI: 0.317<ICC<0.713), F(49,50) = 3.39, P≪0.001).

We used REML estimation in SAS Procedure Mixed (SAS Institute, Cary, NC) to perform mixed-model ANOVA. Main fixed effects were linetype (HR vs. C), sex, and the sex-by-linetype interaction (generation was included as a nuisance variable). Random effects were family nested within line, replicate lines nested within linetype, and the sex-by-line(linetype) interaction term (the number of families in each of the eight lines was 32, 39, 27, 34, 28, 33, 28 & 28 for lines 1 through 8 respectively; note lines designated 1, 2, 4 & 5 were control lines, while 3, 6, 7 & 8 were selected lines). We also separated HR and C lines for two-way ANOVAs of sex and line, both treated as fixed, with family as random nested within line (see Fig. 2). Two-tailed P values are presented.
